# Recent Progress in Dermatology

**Published:** 1886-08

**Authors:** H. J. Reynolds


RECENT PROGRESS IN DERMATOLOGY.

Henry J. Reynolds, M. D., Professor of Dermatology in the College of Physicians and Surgeons, of Chicago, in his annual address as Chairman of the Committee on Dermatology in the Illinois State Medical Society, which convened at Bloomington, May 18, 1886, said:

"It is difficult if not impossible to determine in a year's time whether certain advances and discoveries in this department, as in other departments of medical science, are really such or are only so-called. It would not, however, be speaking in very flattering terms of the intelligence of the writers of the vast array of literature that is yearly presented to the medical profession, nor of those who eagerly peruse the same, were we to assume that even only a little in the direction of advancement had been presented. While, however, but comparatively little perhaps in this department has been presented during the past year, that can in every respect be regarded as real advancement, very many important clinical facts and experiences have nevertheless been presented, which, in addition to establishing new theories, serve the equally important purpose of tending to show the fallacy of old ones. Therefore, not presuming to present all that has appeared on record in this light during the past year, I shall ask the indulgence of the SOCIETY while I enumerate such as have come to my notice or been deemed worthy of mention, together with some personal remarks regarding the same, and leave the matter as regards the determination of the truthfulness and value of the so-called advances for further investigation and your wise judgment to decide."

He then referred to the grouping together of several rare conditions by Duhring under the one general head of what he called dermatitis herpetiformis, and to certain other changes that had been recently made in the nomenclature ot skin diseases.

He next, in his remarks on acne, spoke of the procedure recently proposed by Sherwell, of Brooklyn, for that disease, viz., the passing of sounds in the male urethra; and cited the cases more recently reported by Denslow, of St. Paul, as corroborative of the beneficial results of this supplementary measure in the treatment of acne. For the deep or indurated form of this disease he spoke of the method which he himself usually adopted, and which he believed was original with himself. He always lances those apparently papular indurations deeply, and invariably finds pus at the bottom, with a tendency to burrow still deeper, rather than come to the surface. He then, to prevent the wound from healing over on the surface—which it is otherwise sure to do, and leave the miniature subcutaneous abscess to go on as before, and remain indefinitely—passes a probe dipped in carbolic acid to the bottom and fills the whole incision from bottom to top with a small piece of absorbent cotton and leaves it to granulate from the bottom, which it will never fail to do, leaving only an almost invisible scar.

He referred to a suggestion made by Fox, of New York, as to the possible, if not probable, cure of leprosy, if the proper moral effect could be brought to bear upon those patients, stating that the moral effect was usually depressing in the extreme, banished as they usually are from society, and imprisoned for life, as it were, with only the promise of a most miserable existence and a lingering death.

He referred briefly to the pigmentary syphilide and to a case reported by Dr. R. W. Taylor, of New York, and one by himself of this condition.

He then cited the treatment suggested by Bulkley for carbuncle without incision and poultice, viz: an ointment containing ergot and oxide of zinc locally, and sulphide of calcium and saline laxative with iron internally. He also spoke of the treatment so highly recommended by Dr. Hibberd, of Richmond, Ind., of applying with friction every three hours oleate of morphine.

He then, in speaking of urticaria, said that Lassar claimed to have reduced the frequency and cut short the duration of violent attacks of this disease by giving twenty-four grain doses of salicylate of sodium repeated every two hours until three doses have been taken.

In ringworm of the scalp Alder Smith recommended seven grains of chrysarobin to an ounce of chloroform, his theory being that the chloroform dissolved the sebaceous and fatty substances away, and favored thereby the penetration of the chrysarobin. A. J. Harrison, he said, for this affection, applies first a mixture of one-half a drachm of iodide of potassium to an ounce of liquor potassii. When this has penetrated he applies a solution composed of three grains of bichloride of mercury to the ounce of sweet spirits of nitre; his theory is that the liquor potassii softens the hair bulbs and facilitates the penetration of the iodide of potassium. Then when the mercury mixture is applied the biniodide is formed at the bottom of the follicle, a chemical change which he claims to be very beneficial.

The writer then referred to the use of chrysarobin internally, so highly recommended by Stocquart a short time ago for various skin diseases. For theoretical reasons and from experience he did not believe the drug had any special value as an internal remedy; and even the original advocate did not, of late, claim so much for it as formerly.

Brief reference was then made to the use of cocaine in dermatological practice. He said he had found it to be of some service to allay itching in certain cases of eczema and in pruritis ani, in strong solution. He had used it to relieve the pain incident to the removal of superfluous hairs by electrolysis, but the remedy was not sufficiently absorbed by the skin, as a rule, to be very beneficial.

